# Linkage mapping aided by *de novo* genome and transcriptome assembly in *Portunus trituberculatus*: applications in growth-related QTL and gene identification

**DOI:** 10.1038/s41598-017-08256-8

**Published:** 2017-08-11

**Authors:** Jianjian Lv, Baoquan Gao, Ping Liu, Jian Li, Xianliang Meng

**Affiliations:** 1 Key Laboratory of Sustainable Development of Marine Fisheries, Ministry of Agriculture, Yellow Sea Fisheries Research Institute, Chinese Academy of Fishery Sciences, 266071 Qingdao, China; 2 Laboratory for Marine Fisheries and Aquaculture, Qingdao National Laboratory for Marine Science and Technology, No. 1 Wenhai Road, Aoshanwei Town, Jimo, 266237 Qingdao China

## Abstract

A high-resolution genetic linkage map is an essential tool for decoding genetics and genomics in non-model organisms. In this study, a linkage map was constructed for the swimming crab (*Portunus trituberculatus*) with 10,963 markers; as far as we know, this number of markers has never been achieved in any other crustacean. The linkage map covered 98.85% of the whole genome with a mean marker interval of 0.51 cM. The *de novo* assembly based on genome and transcriptome sequencing data enabled 2,378 explicit annotated markers to be anchored to the map. Quantitative trait locus (QTL) mapping revealed 10 growth-related QTLs with a phenotypic variance explained (*PVE*) range of 12.0–35.9. Eight genes identified from the growth-related QTL regions, in particular, RE1-silencing transcription factor and RNA-directed DNA polymerase genes with nonsynonymous substitutions, were considered important growth-related candidate genes. We have demonstrated that linkage mapping aided by *de novo* assembly of genome and transcriptome sequencing could serve as an important platform for QTL mapping and the identification of trait-related genes.

## Introduction

*Portunus trituberculatus* (Crustacea: Decapoda: Brachyura), commonly known as the swimming crab, is naturally distributed in the coastal waters of China, Japan, Korea and other East Asian countries^1^. The crab has become one of the most important economic species for its high nutritional value and fast growth in marine aquaculture^2^. In 2015, the total production of the swimming crab was 117,772 tons in China; it is a dominant crustacean species in mariculture.

Because of its important economic status, great efforts have been made to check the genome architecture of the swimming crab, including cDNA library construction^3, 4^, transcriptome sequencing^1, 5^, molecular marker development^6–8^, and genetic-linkage map construction^9^. In order to comprehensively elucidate genomic characteristics, high-resolution linkage maps and whole genome sequencing are necessary for this species. However, due to large numbers of chromosomes and complex genomes, it is usually difficult to construct high-resolution linkage mapping and carry out whole genome sequencing for many crustacean species^10, 11^. To date, a draft genome has been reported for only one economically important crustacean species, *Eriocheir sinensis* ^12^.

A high-resolution genetic linkage map is an essential tool for decoding genetics and genomics, such as QTL fine-mapping for important economic traits, gene map-based cloning, assisting with genome assembly, and comparative genome analysis between different species^10, 13^. An accurate high-resolution genetic linkage map is also an important foundation to the genetic breeding of a species, and is indispensable for marker-assisted selection (MAS) of breeding^14, 15^. Although accurate high-density linkage mapping is relatively straightforward for many organisms, it is usually difficult for crustacean species, especially decapods, because of their high number of chromosomes^11^. Previous genetic maps of crustaceans have been successfully obtained and used to map QTLs for economic traits^9, 16–19^; however, the number of available markers were restricted from hundreds to a few thousand, which made it difficult to carry out fine-scale QTL mapping for important traits.

With the development of next-generation sequencing (NGS) and restriction-site-associated DNA (RAD) sequencing technology, some crustacean linkage maps have been constructed with thousands of markers and average marker distances <1 cM, such as *Penaeus monodon* and *E*. *sinensis* ^11, 20^. The first genetic map of *P*. *trituberculatus* have been constructed using simple sequence repeat (SSR) markers and amplified fragment length polymorphism (AFLP) techniques. However, the resolution of these maps is relatively low (with a mean marker interval of 8.7 cM), limiting their usefulness in QTL mapping and genome assembly. Thus, accurate linkage mapping with more markers of *P*. *trituberculatus* is urgently required. Recently, a method of *de novo* SNP discovery and genotyping called specific length amplified fragment sequencing (SLAF-seq) was reported, which was similar with RAD technology^21^. Based on deep sequencing and double barcode genotyping systems, this method was accurate and cost-effective for linkage map construction. High-density linkage maps for *Litopenaeus vannamei*, a dominant crustacean species in global seafood mariculture with complexity genome, have been successful constructed using the SLAF-seq.^10^, which provides a very valuable reference for the genetic linkage map construction of *P*. *trituberculatus*.

The purpose of this study was to construct a genetic linkage map for QTL mapping and assist with further genome assembly in *P*. *trituberculatus*. Firstly, genome survey analysis was carried out to investigate the basic genome characteristics. Secondly, a linkage map was constructed via SLAF-seq. Thirdly, QTLs of growth traits and growth-related genes were identified based on the integration of linkage map, genomic scaffolds/contigs and transcriptome unigenes.

## Materials and Methods

### Genome survey analysis and *de novo* assembly

A male crab from the ninth generation full sib family (F9) was used in genome survey analysis in Beijing Biomarker Technologies Co., Ltd. (Beijing, China). Muscle tissue was used to extract DNA. Four paired-end libraries with insert size of 270 base pairs (bp) on average were constructed from randomly fragmented genomic DNA. Sequencing was performed on the Illumina HiSeq. 4000 sequencing platform with a 151 bp read length, and almost all the insert fragments could be sequenced completely by both paired-end sequencing. Clean reads were obtained after filtering and correction of the sequence data, and were relatively accurate for estimating the size of the genome and heterozygosis.

SOAPdenovo software (http://soap.genomics.org.cn/soapdenovo.html) were applied for *de novo* genome assembly with the following parameters: the k value in K-mer was set at 95. The usable reads >200 bases in length were selected to realign the contig sequences because the sequences <200 bp were likely to be derived from repetitive or low-quality sequences. Then, the paired-end relationship between reads was coincident between contigs. The scaffolds were constructed step by step using insert size paired-ends.

Due to the relatively low conservatism of the repetitive sequence among species, a specific repetitive sequence database was built to predict repeat sequences. The software programs LTR_FINDER^22^, MITE-Hunter^23^, RepeatScout^24^, and PILER-DF^25^ were used to construct a *de novo* repeat library, classified by PASTEClassifier^26^, and combined with the Repbase transposable element library to act as the final library^27^. The software Repeat-Masker was then run to find homologous repeats in the final library^28^. Then, based on K-mer analysis, information on peak depth and the number of 21-mers was obtained. Its relationship was expressed by using the following algorithm: Genome size = K-mer num/Peak depth.

After filtering scaffolds of <200 bp in size, three strategies were used for *de novo* gene prediction and annotation. Firstly, SNAP^29^ and Augustus^30^ were used for *de novo* gene prediction with parameters trained on Portunidae. Secondly, GeneWise^31^ was used to predict genes based on homologous proteins. Thirdly, PASA^32^ was used to predict genes based on predicted unigenes from transcriptome data. Finally, the results obtained by the three strategies were integrated by EVM^33^.

### Mapping population

The F1 full-sib family for linkage map construction was created by artificial insemination using a male parent from a F9 full sibling and a female parent from the wild population of the Bohai Sea, China. Then the mapping population was reared in Chang-Yi AquaFarming Company of Weifang in 2015. A total of 120 progenies were randomly selected at harvest time (four individual marker genotypes that do not conform to Mendel’s law of inheritance were excluded from the follow-up study). Five growth traits including body weight (*BW*), full carapace width (*FCW*), carapace width (*CW*), carapace length (*CL*) and body height (*BH*) were measured for each individual. Muscle tissuses were sampled and immediately preserved in liquid nitrogen. Genomic DNA were extracted using TIANamp Marine animal DNA extraction kit (Catalog Number: DP324, TIANGEN, Beijing, China). The DNA concentration was determined using a NanoDrop 1000 Spectrophotometer (NanoDrop, Wilmington, DE, USA). DNA integrity was evaluated via electrophoresis in 1% agarose gel.

### SLAF sequencing

The way of SLAF sequencing (specific-locus amplified fragment sequencing) library construction and sequencing follows the previously described method with minor modification^21^. Unlike in the literature^21^, in this research, genome DNA was incubated at 37 °C with one restriction endonucleases  RsaI (New England Biolabs, NEB) in one digestion step. A single-nucleotide (A) overhang were added to the digested fragments with Klenow Fragment (NEB) and dATP at 37 °C, and then the Duplex Tag-labeled Sequencing adapters (PAGE purified, Life Technologies, Gaithersburg, MD, USA) were ligated to the A-tailed DNA with T4 DNA ligase. The PCR reaction was performed using diluted restriction-ligation samples, dNTP, Q5® High-Fidelity DNA Polymerase and PCR primers: AATGATACGGCGACCACCGA and CAAGCAGAAGACGGCATACG (PAGE purified, Life Technologies). The PCR productions were purified using Agencourt AMPure XP beads (Beckman Coulter, High Wycombe, UK) and pooled. The pooled sample was separeted by electrophoresis in a 2% agarose gel. Fragments with 400–450 bp (with indexes and adaptors) in size were excised, purified using QIAquick Gel Extraction Kit (QIAGEN). The gel-purified product was sequenced using the paired-end sequencing method on the Illumina HiSeq. 2500 system (Illumina, Inc; San Diego, CA, U.S.) according to the manufacturer’s recommendations. The read length was 150 bp.

### Marker discovery and genotyping

SLAF marker identification and genotyping were performed using procedures described by Sun *et al*.^21^. Briefly, low-quality reads with Q (quality score) <20e (‘e’ represents base sequencing error rate, Q = −10*log10e) were filtered out and then raw reads were sorted to each progeny according to duplex barcode sequences. After the barcodes and the terminal 5-bp positions were trimmed from each high-quality read, clean reads were clustered by similarity above 90%. Sequences clustered together were defined as one SLAF locus^34^. Single nucleotide polymorphism (SNP) loci of each SLAF locus were then detected between parents, and SLAFs with more than eight SNPs were filtered out to avoid affecting the accuracy of the genetic linkage map, because such regions were considered to be the high frequency variability regions. Alleles of each SLAF locus were then defined according to parental reads, while for each offspring the reads were used to define alleles. Only SLAFs with two to four alleles were identified as polymorphic and considered potential markers. Based on SNP genotypes of SLAF marker, polymorphic markers could be converted to different segregation patterns, and the five patterns (ab × cd, ef × eg, hk × hk, lm × ll, nn × np) could be used in linkage map construction for the full-sib family. All polymorphic SLAFs loci were genotyped with consistency in the parental and offspring SNP loci. Genotype scoring was then performed using a Bayesian approach to further ensure the genotyping quality^21^.

### Genetic linkage map construction

To ensure efficient construction of the high-quality map, a newly developed HighMap strategy was utilized to order markers and correct genotyping errors within LGs^35^. Marker loci were partitioned primarily into linkage groups (LGs) by the modified logarithm of odds (MLOD) scores >5. Firstly, recombinant frequencies and LOD scores were calculated by two-point analysis, which were applied to infer linkage phases. Then, enhanced gibbs sampling, spatial sampling and simulated annealing algorithms were combined to conduct an iterative process of marker ordering^36, 37^. Once a stable map order was obtained after 3–4 cycles, we turned to the next map building round. A subset of currently unmapped markers was selected and added to the previous sample with a decreased sample radius. The mapping algorithm was repeated until all the markers were mapped appropriately. The error correction strategy of SMOOTH was then conducted according to the parental contribution of genotypes^38^, and a k-nearest neighbor algorithm was applied to impute missing genotypes^39^. Map distances were estimated using the Kosambi mapping function^40^.

The estimated genome size (Ge) was calculated using two methods described by Yu *et al*.^10^. Briefly, the average marker interval(s) was calculated by dividing the total length of each linkage group by the number of intervals. Genome estimation size 1 (Ge1) was calculated by adding 2 s to the length of each linkage group^41^. Genome estimation size 2 (Ge2) was determined by taking the total length of the linkage groups multiplied by the factor (m + 1)/(m − 1), where m is the number of loci on each linkage group^42^. The average of the two estimated genome sizes was the final estimated genome size. The observed genome length was the summed length of all linkage groups. The map coverage was calculated by observed genome length/the estimated genome size.

The method used to estimate the ratio of female/male recombination rate has been previously described^10^. Briefly, the common informative markers between the female and male maps were extracted. The map length for each linkage group containing the common markers was calculated separately. Sex differences were represented by the female map length divided by male marker length between the common markers.

### Integration of linkage map, genomic scaffolds/contigs and transcriptome unigenes

The reference transcriptome was *de novo* assembled using the raw reads from our previous work^1, 5, 43^. The clean reads were assembled by Trinity as described previously^44^, followed by TIGR Gene Indices clustering tools (TGICL)^45^. The longest assembled sequences were referred to as contigs. The reads were then mapped back to contigs with paired-end reads to detect contigs from the same transcript and the distances between these contigs. Finally, sequences were obtained that lacked Ns (reads with ambiguous bases N) and could not be extended on either end [30]. Such sequences were defined as unigenes. The unigenes were annotated via public databases by BlastX via an E-value cut-off of 1.0 × 10^−5^. Public databases used in this study included the GenBank nr database hosted by NCBI (http://www.ncbi.nlm.nih.gov/), the Swiss-Prot database (http://web.expasy.org/docs/swiss-prot_guideline.html), the Kyoto Encyclopedia of Genes and Genomes (KEGG) and the Clusters of Orthologous Groups (COG) (http://www.ncbi.nlm.nih.gov/COG/).

An integration map was constructed by integrating the linkage map markers, transcriptome unigenes and genomic scaffolds/contigs mainly via BLAT and the Circos tool^46^. Before BLAT analyzing, the software Repeat-Masker was run to mask homologous repeats in order to increase the accuracy of alignment^28^. In a brief statement, the three sets of data were integrated when the sequence could be aligned to each other via BLAT analysis. The outer ring of the integration map represents the physical distance of each linkage group, which was the sum of the length of the genomic scaffold/contig sequence alignment to the linkage group via BLAT analysis with the default parameters (minMatch 2, minScore 30, minIdentity 90, maxGap 2). The intermediate ring represents the alignment relation between markers and the scaffold/contig sequences. If the two ends of a marker matched different scaffold/contig sequences, then this marker was linked to the two scaffold/contig sequences. The inner ring represents the alignment relation between transcriptome unigene and the scaffold/contig sequences, and calculates the number of unigenes per kb of scaffold/contig sequence.

### QTL mapping and growth-related genes identified

The phenotypic data are given in Supplementary Table S1. QTL analysis was carried out using the interval mapping method and multiple-QTL model mapping (MQM) methods in the program MapQTL 4.0 software as described^47, 48^. Composite interval mapping (CIM) was adopted with 1 cM walking speed. Two-LOD support intervals were constructed as 95% confidence intervals^49^. Likelihood-ratio statistic (LOD) with a minimum score of 3.0 was used to declare the presence for each significant QTL in a particular genomic region, which was determined using 1,000 permutations. The phenotypic variance explained (*PVE*) was also calculated in MapQTL4.0 based on the population variance in the mapping population.

The genes located in the QTL regions were considered growth-related genes, which were identified by the integrated map and QTL mapping. Firstly, by finding the markers in the QTL regions. Secondly, to verify whether these markers could be compared to the genomic scaffolds/contigs or transcriptome unigenes. And thirdly, to check whether these sequences could be explicitly annotated via blastx with public databases.

## Results

### Genome survey analysis of *Portunus trituberculatus*

**Figure 1:**
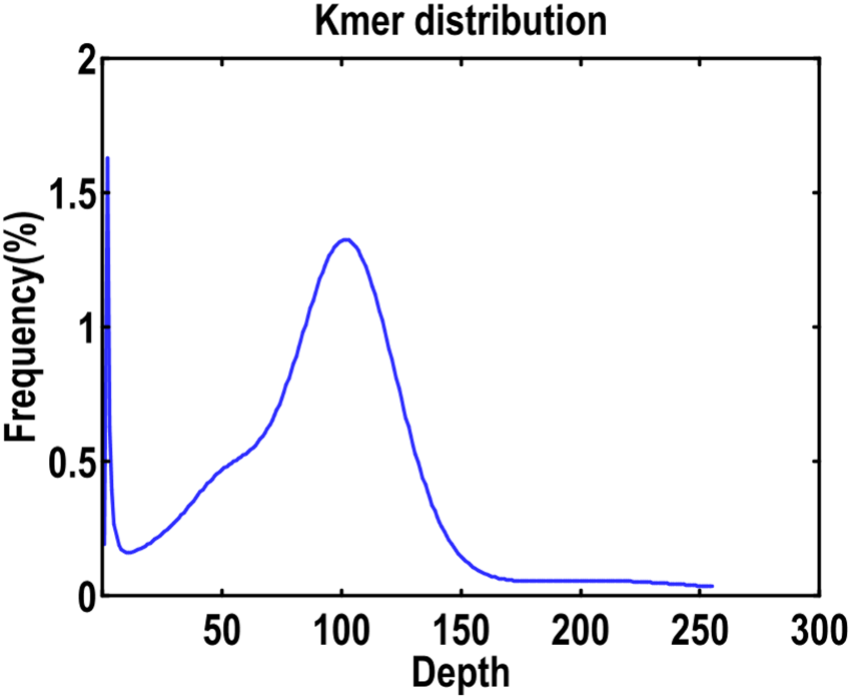
K-mer frequency distribution of sequencing reads.

Four 270 bp short paired-end DNA libraries were constructed for the genome survey analysis. After sequencing, a total of 113.83 Gb high-quality reads with 41.04% GC content were obtained, which covered approximately 141.24-fold the genome size of *P*. *trituberculatus*. Sequencing raw data have been submitted to the Sequence Read Archive (SRA) database of NCBI with the accession code SUB2680377. A K-mer curve was constructed based on the frequencies of 21-mers (nucleotide strings with a length of 21 bp) among the raw sequencing data (Fig. 1). K-mer analysis revealed that there was a peak at the K-mer depth of 101. Genome size was estimated as 805.92 Mb with remarkably high heterozygosity (0.96%), which was occupied by approximately 39.42% repeat sequences (Table 1). A total of 1,268,724 contigs with an N50 size of 756 bp were obtained, which covered 833,944,844 bp of the genome. Scaffolds with N50 of 1154 bp were also generated (Table 1). Although the N50 size is smaller, it covered more than 90% of the transcriptome unigenes via blast analysis (Table 2). Based on the sequences of scaffolds/contigs, we finally predicted a gene set containing 15,267 genes (Table S2).

**Table 1:** Statistics of the the genome survey and assembly.

Category	
Genome size (Mb)	805.92
heterozygosity	0.96%
repeat sequences	39.42%
Data (Gb)	113.83
Depth (X)	141.24
Number of contigs	1,268,724
Total length (bp) of contigs	833,944,844
Contig N50 (bp)	756
Number of scaffolds	898,300
Total length (bp) of scaffolds	842,129,340
Scaffold N50 (bp)	1154

**Table 2:** Unigenes of transcriptome blast with genomic scaffolds/contigs.

Range of unigene length	Total number	Aligned number	Percentage (%)
All	219,728	199,221	90.67
> = 500	94,110	91,331	97.05
> = 1,000	56,515	55,990	99.07

### SLAF marker and linkage map

**Figure 2:**
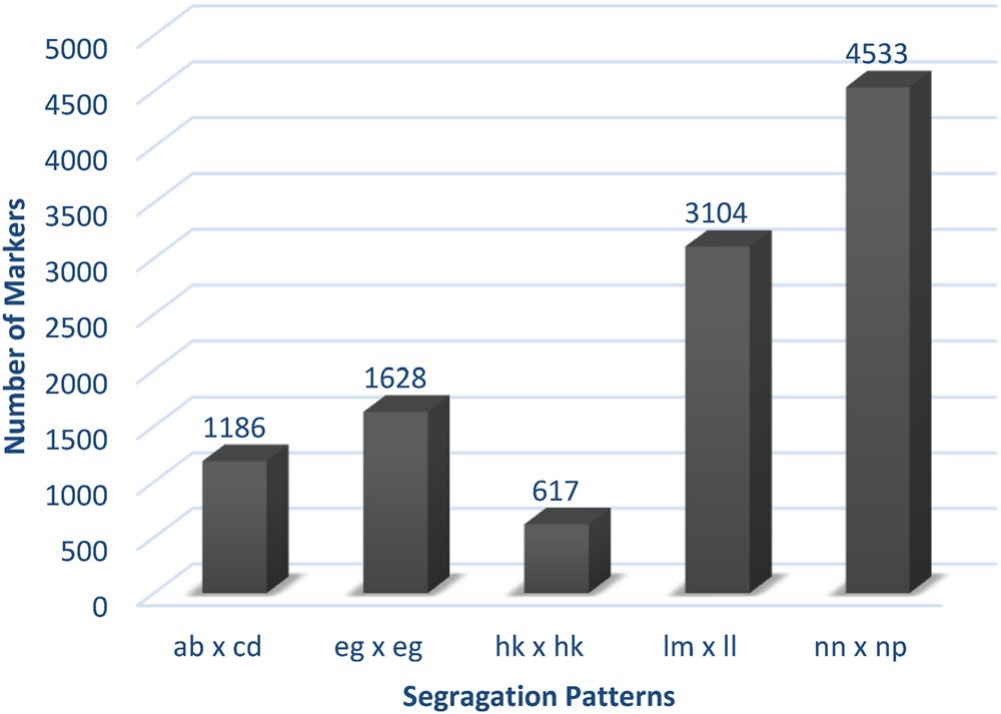
Statistics of genotyped SLAF markers in five segregation patterns.

After SLAF sequencing, 317,918,396 paired-end reads were generated for the mapping family (two parents and 116 progenies). A total of 60,319 polymorphic SLAF markers were identified from 152,449 SLAF markers, of which 11,068 could be successfully genotyped in both parents and offspring (Table 3). SLAF markers with the five segregation patterns (ab × cd, ef × eg, hk × hk, lm × ll, nn × np) could be used in linkage map construction for the full-sib family, of which nn × np was the major pattern (41.0%), followed by lm × 11 (28.0%) (Fig. 2). The average read depth of genotyped markers were 25.10, 74.71 and 100.23 in the offspring, male and female parents, respectively (Table S3).

**Table 3:** Statistics of the SLAF data.

Category	
Number of reads	317,918,396
Number of high quality SLAF	152,449
Polymorphic SLAF	60,319
Average depth in male parent	74.71
Average depth in female parent	100.23
Average depth in offspring	25.10

**Figure 3:**
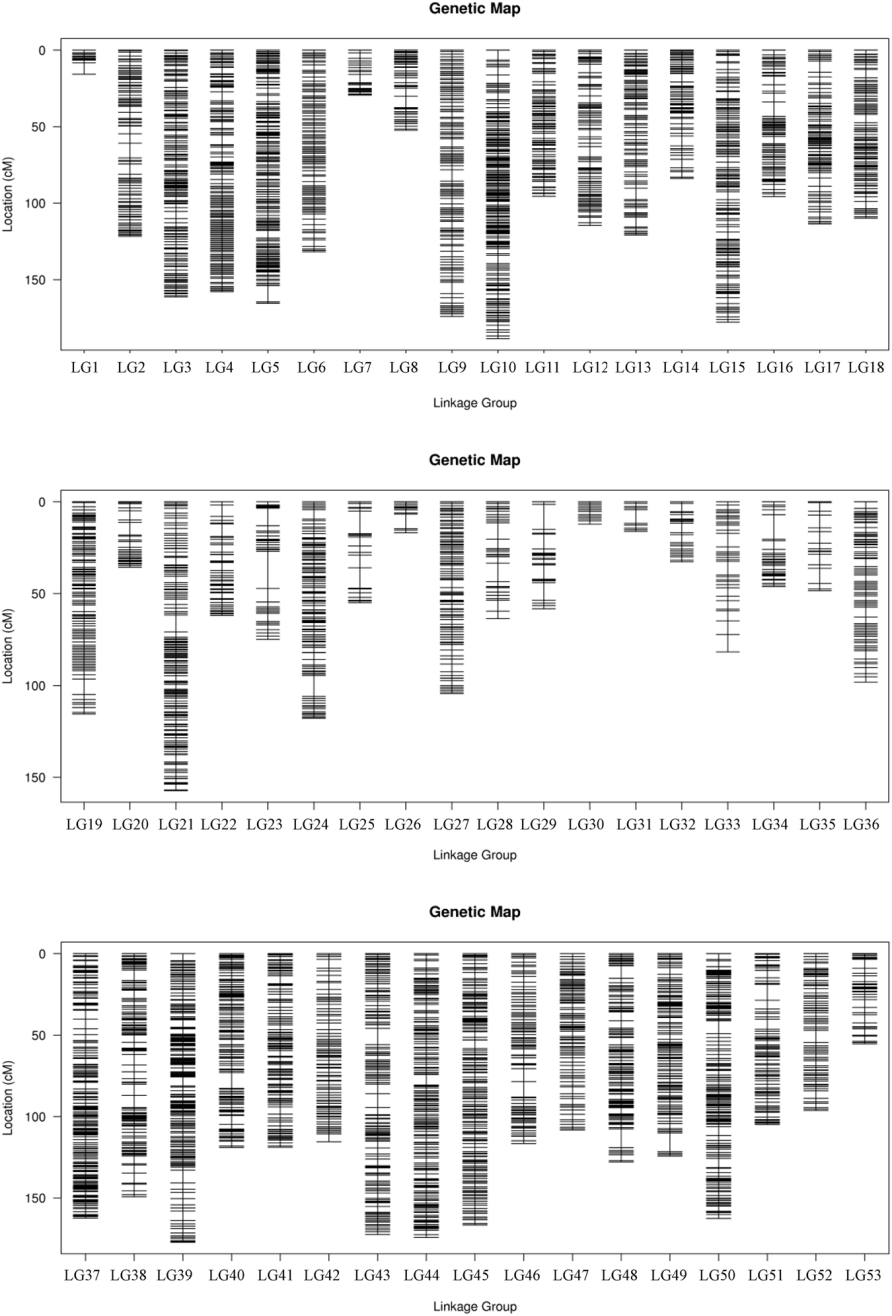
The sex-averaged map of *P*. *trituberculatus*.

A pseudo-testcross strategy was used to construct linkage maps in this study. Finally, we obtained a linkage map containing 53 linkage groups with 10,963 markers (Table 4, Figs 3 and S1–S4, Tables S4, S5 and S6). The total map distance of the sex-averaged map was 5,557.85 cM, which covered 98.85% of the genome based on the estimated total length of the genome map. The distribution of markers among linkage groups was evaluated by statistics of the marker interval. On average, 96% of the sex-averaged map were covered by markers with an interval distance of less than 5 cM and the mean distance between two markers was 0.51 cM (Table S4).

**Table 4:** Summary of *Portunus trituberculatus* linkage map.

Category	sex-averaged map	male map	female map
Number of markers mapped	10,963	7001	7875
Number of linkage group	53	53	53
Minimum length of linkage group (cM)	15.74	5.25	12.21
Maximum length of linkage group (cM)	188.47	192.48	225.12
Minimum markers number per linkage group	21	16	17
Maximum markers number per linkage group	473	322	394
Average marker interval (cM)	0.51	0.64	0.74
Observed genome length (cM)	5557.85	4454.68	5759.27
Estimated genome length (cM)	5622.79	4528.71	5854.63
Genome coverage (%)	98.85	98.37	98.37

The female and male maps exhibited different marker numbers and recombination rates. In general, the female map contained more markers (7,875) than the male map (7,001). The female/male ratio of recombination frequency was 1.47 over all the linkage groups (Table 5). However, the ratio differed between the groups. The ratio ranged from 0.48 to 8.81 among the 53 linkage groups. In 16 of 53 linkage groups, a higher recombination rate was observed in male maps. In 33 of 53 linkage groups, higher recombination rates were observed in the female map. In the other four linkage groups, the recombination rates were the same between females and males.

**Table 5:** Recombination rates in male and female maps.

LG ID	Female	Male	Female/Male	LG ID	Female	Male	Female/Male
LG1	6.118	3.493	1.75	LG28	52.591	73.017	0.72
LG2	127.063	109.812	1.16	LG29	56.459	60.121	0.94
LG3	174.886	145.993	1.20	LG30	12.205	12.205	1.00
LG4	100.168	58.179	1.72	LG31	16.148	16.148	1.00
LG5	151.46	142.515	1.06	LG32	6.496	13.396	0.48
LG6	131.702	131.702	1.00	LG33	2.556	5.248	0.49
LG7	5.972	8.79	0.68	LG34	59.825	18.402	3.25
LG8	68.981	7.826	8.81	LG35	35.745	61.194	0.58
LG9	174.021	174.021	1.00	LG36	30.031	24.819	1.21
LG10	182.233	108.927	1.67	LG37	147.01	177.796	0.83
LG11	103.878	78.34	1.33	LG38	158.546	140.024	1.13
LG12	117.295	101.038	1.16	LG39	208.809	145.493	1.44
LG13	31.878	31.488	1.01	LG40	103.903	130.615	0.80
LG14	44.862	37.284	1.20	LG41	122.444	106.463	1.15
LG15	140.365	175.674	0.80	LG42	54.656	37.358	1.46
LG16	84.59	87.304	0.97	LG43	183.305	158.005	1.16
LG17	48.253	31.598	1.53	LG44	163.434	151.454	1.08
LG18	118.477	88.634	1.34	LG45	169.153	160.574	1.05
LG19	59.004	35.977	1.64	LG46	88.174	53.638	1.64
LG20	31.225	36.768	0.85	LG47	70.878	46.405	1.53
LG21	169.186	145.405	1.16	LG48	98.241	115.086	0.85
LG22	55.948	50.28	1.11	LG49	123.014	97.074	1.27
LG23	16.905	31.768	0.53	LG50	146.354	162.827	0.90
LG24	102.627	105.335	0.97	LG51	109.972	98.068	1.12
LG25	49.094	57.48	0.85	LG52	96.849	85.126	1.14
LG26	22.347	11.43	1.96	LG53	63.637	46.974	1.35
LG27	84.908	70.677	1.20	Total	2403.446	2067.738	1.47

### Linkage map integration

**Figure 4:**
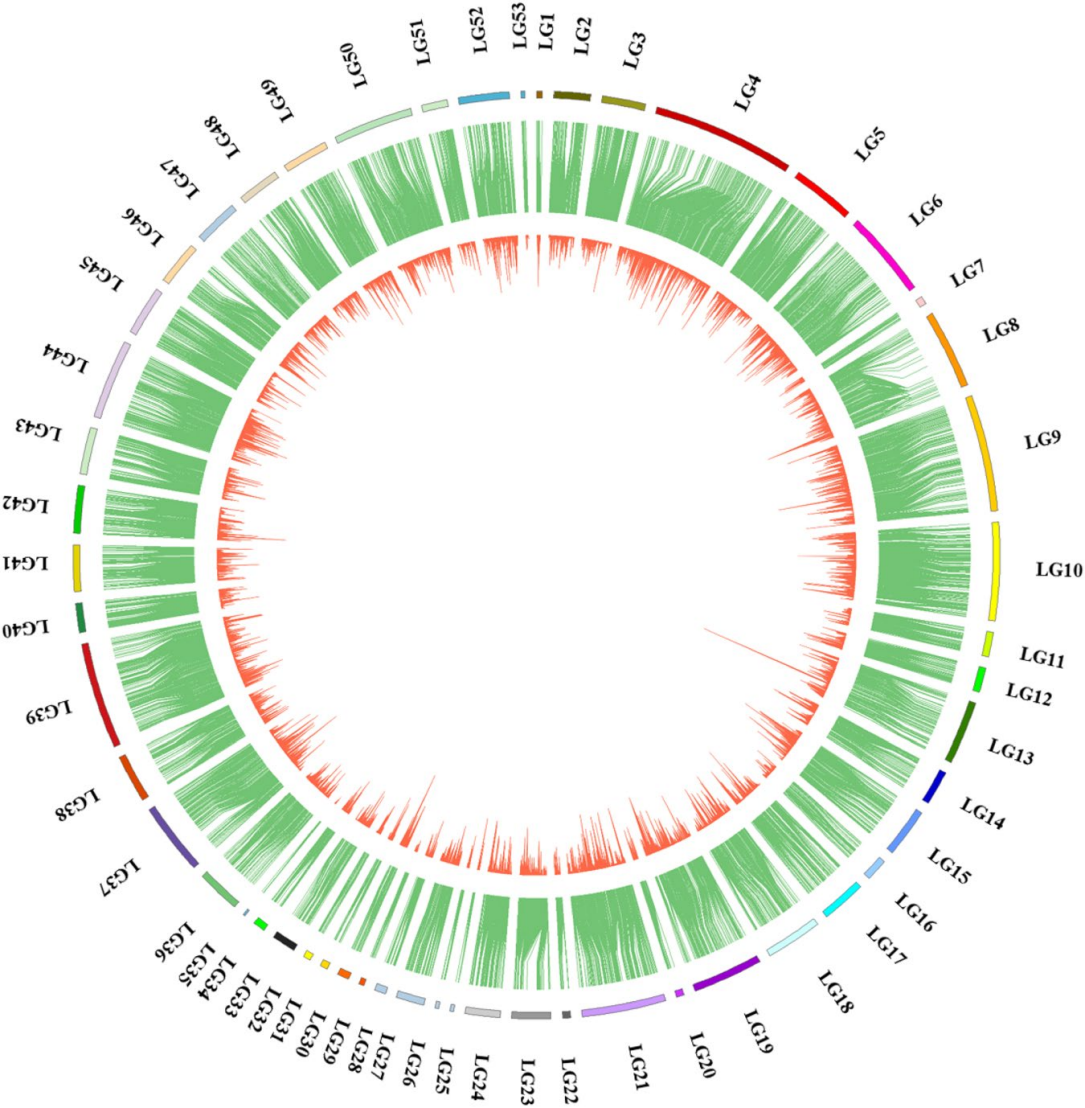
Integration of linkage map, genomic scaffolds/contigs and transcriptome unigenes. Outer ring, the linkage group; Intermediate ring, contigs or scaffolds of genome assembly aligned with markers from the linkage map; Inner ring, unigene sequences of transcriptome aligned with scaffold/contig sequences.

Of the 10,963 markers in the map, 5,266 markers were able to align to 5,085 genomic scaffolds/contigs with high confidence (both ends of the marker could be matched to the same scaffold) (Table S7). The transcriptome is another important genomic resource. In previous work, 152,596 unigenes were assembled based on all Illumina reads. A total of 4,675 markers were homologous to the transcriptome unigenes with an average length of 2,112 bp, which could be linked with the linkage map (Table S8). Based on information from blast, the markers of linkage maps, genomic scaffolds/contigs and unigenes of transcriptome could be integrated (Fig. 4). Finally, a total of 7,487 markers could be aligned to the genomic scaffolds/contigs or transcriptome unigenes after integrating, of which 2,378 markers could be explicitly annotated via blastx with the public Nr and Swissprot databases.

### QTL mapping of growth traits

**Figure 5:**
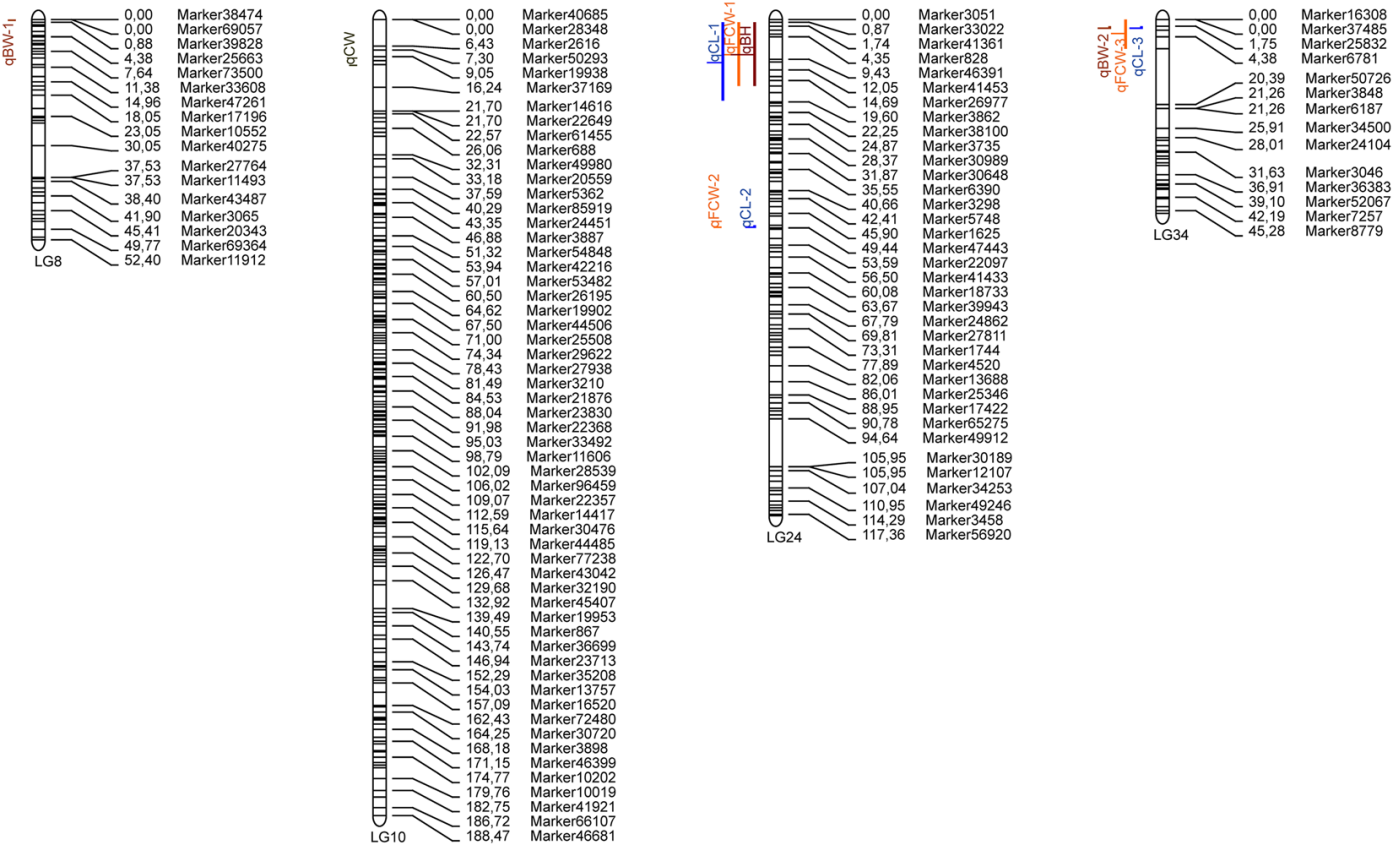
QTL mapping of growth traits. QTLs of different traits is represented by different colors.

On the basis of the map, a total of ten QTLs were detected for all the five growth-related traits by MapQTL 4.0 software, which were distributed on LG8, LG10, LG24 and LG34 (Table 6 and Fig. 5). These QTLs with LOD values of 3.03–6.91 contributed to *PVE* of 12.0%–35.9%. Among them, *qBH* located at 0.87–16.015 cM of LG24 with the highest LOD score of 6.91, and correspondingly had the highest *PVE* value of 35.9%. Some QTL intervals were clustered together on their respective LGs. One major cluster containing three QTLs (*qFCW*-*1*, *qCL*-*1* and *qBH*) was dectected between the positions of 0.870–19.597 cM on LG24. On LG34, another cluster situated within a short region (0–7.034 cM) also consisted of three QTLs (*qBW*-*2*, *qFCW*-*3* and *qCL*-*3*). Forty-eight markers were located in the QTL intervals, among which, thirty-three markers (68.8%) distributed on LG24 (Table S9).

**Table 6:** Characteristics of growth related QTLs.

Trait	QTL	Linkage Group	Start (cM)	End (cM)	Marker Number	Max LOD	Max PVE
BW	qBW-1	8	0	0	5	3.15	12.3
	qBW-2	34	1.755	2.624	3	3.22	16.4
FCW	qFCW-1	24	0.870	16.015	17	3.75	20.3
	qFCW-2	24	49.437	49.437	1	3.03	12.0
	qFCW-3	34	0	7.034	9	3.81	21.2
CW	qCW	10	10.944	10.944	1	3.10	12.2
CL	qCL-1	24	0.870	19.597	26	4.14	22.0
	qCL-2	24	49.002	49.872	7	3.27	12.8
	qCL-3	34	1.755	2.624	3	3.27	17.2
BH	qBH	24	0.870	16.015	17	6.91	35.9

### Growth-related gene identification

**Figure 6:**
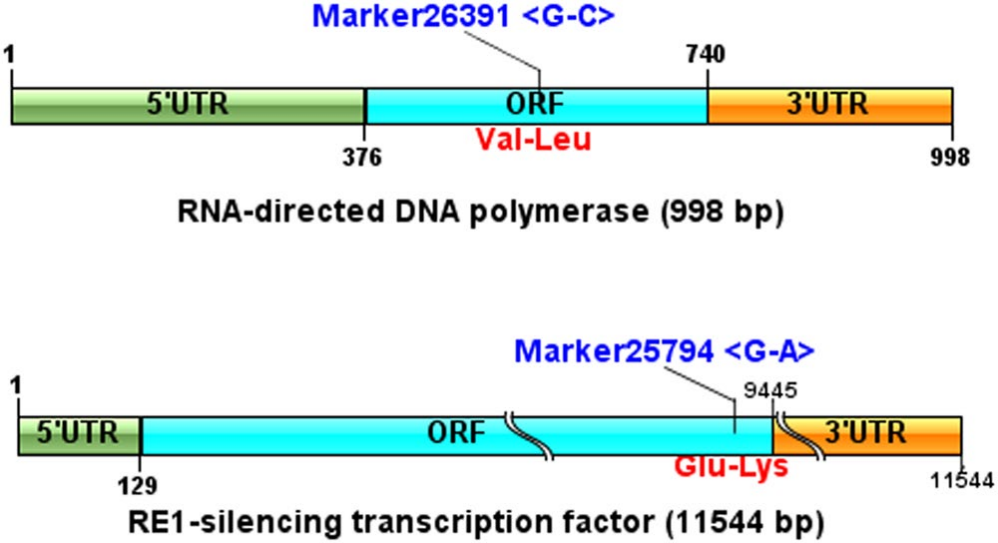
Structural features and nonsynonymous sites of REST and RNA-directed DNA polymerase genes. cDNA sequences of two genes were assembled based on transcriptome unigene data. Marker26391 with a SNP (G–C) on RNA-directed DNA polymerase led to the nonsynonymous change of Val to Leu; Marker25794 with a SNP (G–A) on REST led to the nonsynonymous change of Glu to Lys.

Based on the integrated map, 34 of 48 (70.8%) associated markers could be aligned to the genomic scaffolds/contigs (20) or transcriptome unigenes (24) (Table S9). Among which, eight aligned sequences could be explicitly annotated via blastx with public databases, including RNA-directed DNA polymerase, xylulose kinase, monocarboxylate transporter, glycoprotein 6-alpha-L-fucosyltransferase, mitogen-activated protein kinase, integrin, poly [ADP-ribose] polymerase and RE1-silencing transcription factor. These genes were distributed on two linkage groups, which included five genes located on LG24 and three genes located on LG34. Five genes with markers in exons were considered important growth-related candidate genes (Table 7). In particular, the RE1-silencing transcription factor (REST) and RNA-directed DNA polymerase genes contained nonsynonymous encoded amino acids (Fig. 6), which suggests their potential function in influencing growth regulation.

**Table 7:** Statistics of growth-related genes.

MarkerID	Linkage Group	Position (cM)	Annotation	Marker Location
Marker26271	LG24	2.609	xylulokinase	5′UTR
Marker46391	LG24	9.431	glycoprotein 6-alpha-L-fucosyltransferase	intron
Marker6733	LG24	10.301	Mitogen-activated protein kinase	intron
Marker25749	LG24	13.810	RE1-silencing transcription factor	ORF(nonsynonymous)
Marker10494	LG24	13.810	poly [ADP-ribose] polymerase	3′UTR
Marker26391	LG34	0	RNA-directed DNA polymerase	ORF(nonsynonymous)
Marker25832	LG34	1.755	integrin alpha 8	intron
Marker25048	LG34	2.624	monocarboxylate transporter	5′UTR

## Discussion

*P*. *trituberculatus* is an important marine cultured crustacean species. Given its economic importance, great efforts have been made to clarify the genomic characteristics of this species. However, no high-resolution linkage map and reference genome is yet available for the crab. In this study, a genome survey analysis was carried out and represented the first step towards fully decoding the *P*. *trituberculatus* genome. A accurate linkage map with more than 10 thousand markers was first constructed. QTLs of growth traits and growth-related genes were identified based on the integration of linkage map, genomic scaffolds/contigs and transcriptome unigenes.

### Genome survey analysis

In spite of their economic importance and diverse geographical distribution, little effort has been devoted to decoding crustacean genomes. To date, a draft genome has been reported for only one economically important crustacean species, *E*. *sinensis* ^12^. Although the genome of the crab is highly fragmented with incomplete assemblies (contig N50 size = 6.02 kb), it provides the first glimpse into crustacean genome architecture. In addition, a genome survey analysis has been performed in *L*. *vannamei*, which also highlighted the complexity of the crustacean genome^10^. Thus, to better understand their diversification, phylogeny and molecular mechanisms of economic traits^13^, whole genome sequencing for more crustacean species is indispensable. Our study represents the first step towards fully decoding the *P*. *trituberculatus* genome. Due to the crab with high genomic homozygosity can not be obtained via artificial gynogenesis, we used an individual from the F9 full-sib inbreeding family for genome survey analysis. Compared to *E*. *sinensis* and *L*. *vannamei*, the genome of *P*. *trituberculatus* is smaller and simpler (805.92 Mb) with a lower ratio of repetitive and heterozygous sequences, according to the K-mer analysis; this suggests that genome sequencing and assembling of a crab from successive multi generation full-sib family is relatively feasible. We note that the genome size is inconsistent with previous studies (2.3 pg), in which flow cytometry was used to assess genome size^50^. Flow cytometry has been widely adapted for genome size estimation; although the method is fairly straightforward, the accuracy of estimation is highly dependent on the internal standard and quality of the material used for DNA content measurement^51^. Previous studies in insects have also found that there were slight differences in the genome size assessment from the two methods, and following evidences from whole genome sequencing indicated that k-mer analysis is more accurate^52^. Therefore, we believe the genome size will be accurately calculated after whole-genome sequencing of this species in the future. A total of 1,708,535 contigs with an N50 size of 603 bp were obtained. Although the N50 size is smaller, it covered more than 90% of the transcriptome sequence via blast analysis. We finally obtained a gene set containing 15,267 genes based on the assembled scaffolds/contigs, which is significantly more than the gene number identified for *E*. *sinensis* (7,549)^12^ and horseshoe crab (5,775)^53^.

### Linkage map construction and integration with multiple genomic resources

It is usually difficult to construct high-resolution linkage mapping for crustacean species due to their complex genomes and large number of chromosomes^11^. In this study, taking advantage of SLAF and NGS technologies, 11,068 polymorphic SLAF-tags were successfully identified in both parents and offspring with a high read depth. Previous research showed that the genotyping error rate decreased significantly as read depth increased, and the error rate could almost be ignored when the read depth increased to 12^21^. The average read depth of markers (25.10) was significantly higher than 12 in this study, which ensured a high accuracy for marker genotyping.

Finally, we successfully constructed the linkage mapping including 53 linkage groups, which is consistent with karyotypes of *P*. *trituberculatus* ^54^. The sex-averaged map contained 10,963 markers, which to our knowledge, has never been achieved in any other crustacean. The linkage map covered 98.85% of the whole genome with a mean marker interval of 0.51 cM. The mapping resolution was higher than the map of *P*. *monodon* (0.9 cM) and *L*. *vannamei* (0.7 cM)^10, 20^, and similar to *E*. *sinensis* (0.49 cM)^11^. In addition, each marker developed in this study contained a 200 bp genome sequence (two terminal sequences), which provides sufficient sequence information for trait-related gene identification and comparative genome analysis.

It is important to know the female/male ratio of recombination for both sexes of any species^55^. Based on the length of the common intervals, the recombination ratio between the female and male parents of *P*. *trituberculatus* was 1.47, which was different from the first map (1.01)^9^. These conflicting results may have been derived from the marker types and marker density^10^. Compare to our work, the previous linkage map was constructed mainly using co-dominant AFLP markers and the number of markers was relatively small, which could have influenced the accurate estimation of the female/male ratio of recombination. It is common to find the recombination rate is more restricted in males than in females in the XY sex determination system. For instance, the female to male recombination ratios have been recorded as 8.26 in Atlantic salmon (*Salmo salar*)^56^, 3.25 in rainbow trout (*Oncorhynchus mykiss*)^57^ and 2.0 in grass carp (*Ctenopharyngodon idella*)^58^. However, it’s not always the case in crustaceans. For instance, the female to male recombination ratios was no difference (1.05) in Chinese mitten crab (*Eriocheir sinensis*), an important ecological and economic species with ZW sex determination system^11^. Therefore, whether the restricted male recombination rates shown in this study is related to sex still needs further study.

Integration of a genetic linkage map with scaffolds/contigs obtained from *de novo* genome assembly and transcriptome unigenes will be helpful for anchoring genes to a map; at the same time, they can also indirectly verify the accuracy of the genetic linkage map. A total of 172 scaffolds/contigs containing at least two tightly-linked markers on the genetic linkage map were found in this study (Table S10), and we did not find any scaffold/contig matched with markers located on different linkage groups or with a larger genetic distance. This result strongly proves the accuracy of the map in this study.

A total of 7,487 markers could be aligned to genomic scaffolds/contigs or transcriptome unigenes, a higher number than for similar studies in *L*. *vannamei* (5,922) and *Chlamys farreri* (2,174)^10, 13^. Among which, 2,378 markers could be explicitly annotated via aligned sequences in blastx with public databases. Therefore, before the completion of the whole genome sequencing in *P*. *trituberculatus*, the integration map could provide useful information for anchoring important genes related to traits.

### QTL mapping of growth traits

Breeders are particularly interested in growth traits due to their highly commercial significance in aquaculture. QTL mapping is a principal application of the genetic linkage map, which represents an efficient method to locate trait-related genes for MAS in genetic breeding^13^. In our previous work, growth-related QTLs were mapped on the first genetic linkage map of *P*. *trituberculatus* ^59^. However, the QTL mapping accuracy was limited due to the relatively low resolution of the map. In addition, it is difficult to anchor growth-related genes because of the larger marker interval and marker type. Here, QTLs for five growth traits (*BW*, *FCW*, *CW*, *CL*, *BH*) were located on the linkage map, which represented the main growth traits of the swimming crab. Finally, ten growth-related QTLs were detected and distributed in four linkage groups (LG8, LG10, LG24 and LG34), which reflects the complexity of these polygenic traits. Four significant QTLs were detected with a *PVE* value higher than 20%, suggesting that these QTLs play major roles in growth. The phenomenon of one trait controlled by a few significant QTLs with higher *PVE* values was consistent with the characteristics of growth traits, which were generally controlled by several major genes with higher heritability^59, 60^. Interestingly, QTLs of three traits (*FCW*, *CL*, and *BH*) were located in the same interval of LG24 (0.870–19.597 cM), indicating that these traits may be controlled by the same genes.

Anchoring the markers in different versions of genetic linkage map would contribute to consolidate the structural genomic resources of this species. According to the blast result of marker sequence, two of 55 (pot16 and pot53) SSRs in previous work (Liu *et al*., 2012) could be matched to the present map (Marker46391 and Marker16413). Among which, Marker46391 was located in the growth-related QTL interval in our study, however, the corresponding SSR (pot16) was not associated with growth related traits in the previous research^59^. The contradictory results may be due to the difference of mapping population and resolution of the two studies.

### Identification of growth-related genes

Forty-eight markers were located in the QTL intervals, which will be invaluable as a utility for further evaluation by MAS. Based on the integrated map, eight growth-related candidate genes with explicit annotated information were identified. Previous studies have shown that some of these genes play roles in growth regulation in animals or plants. For example, poly [ADP-ribose] polymerase may regulate cell growth by controlling the ER/IGF-1R/PDZK1 axis^61^, and inhibition of the gene may enhance growth of *Arabidopsis thaliana* ^62^. Compared with wild-type animals, monocarboxylate transporter knockin mice (MCT^+/−^) exhibited a reduced food intake and were insensitive to diet-induced obesity, which provides some insights into their putative roles in weight gain regulation^63^. Mitogen-activated protein kinase was found to participate in the inhibition of cell proliferation via inactivation of the mitogen-activated protein kinase signal pathway in hepatocellular carcinoma^64^. Particularly, REST and RNA-directed DNA polymerase genes with nonsynonymous markers in the ORF were considered important growth-related candidate genes in this study. The role(s) of these genes in growth of *P*. *trituberculatus* are worth further evaluation.

## Conclusions

In this study, a genome survey analysis was conducted for *P*. *trituberculatus*, which represents the first step towards fully decoding the *P*. *trituberculatus* genome. A smaller genome with a lower ratio of repetitive and heterozygous sequences was observed in this species. A genetic linkage map with 10,963 markers was constructed via SLAF-seq. To our knowledge, this number of markers has never been reached for any other crustacean species. Ten growth-related QTLs and eight growth-related genes were identified; these will be useful in clarifying the molecular mechanism of growth regulation and MAS for this important aquaculture species. We demonstrate that a linkage mapping aided by *de novo* genome and transcriptome assembly could serve as an important platform for QTL mapping and identifying trait-related genes.

## Electronic supplementary material

Fig S1-S4 Sex-averaged genetic linkage group

Table S1 Phenotype data of sequenced individuals

Table S2 Gene predict of genome assembly sequence

Table S3 The SLAF marker number and the read depth of each genotyped marker in the mapping family

Table S4 Information of linkage map

Table S5 Marker information of sex-averaged linkage map

Table S6 Sequences of the mapped markers

Table S7 Markers anchored to scaffolds

Table S8 Markers anchored to transcriptome

Table S9 Information of 48 markers in the QTL intervals

Table S10 Contigs or scaffolds containing at least two tightly linked markers
