# Supplementary material for: Linkage mapping aided by de novo genome and transcriptome assembly in Portunus trituberculatus: applications in growth-related QTL and gene identification
Source: Sci Rep. 2017 Aug 11;7:7874. doi: 10.1038/s41598-017-08256-8 (PMC5554138; doi:10.1038/s41598-017-08256-8)
Supplement: Supplementary file 1 — Fig S1-S4 Sex-averaged genetic linkage group [file 41598_2017_8256_MOESM1_ESM.pdf]

Linkage mapping aided by *de novo* genome and transcriptome assembly in *Portunus trituberculatus*: applications in growth-related QTL and gene identification

Jianjian Lv [a,b] Baoquan Gao [a,b] Ping Liu [a,b] Jian Li [a,b]* Xianliang Meng [a,b]

[a] Key Laboratory of Sustainable Development of Marine Fisheries, Ministry of Agriculture, P.R.China, Yellow Sea Fisheries Research Institute, Chinese Academy of Fishery Sciences, 266071 Qingdao,China.

[b] Laboratory for Marine Fisheries and Aquaculture, Qingdao National Laboratory for Marine Science and Technology, No. 1 Wenhai Road, Aoshanwei Town, Jimo, Qingdao, China.

* Corresponding author, E-mail: lijian@ysfri.ac.cn

Excel Spreadsheets of Supplementary Table S1-S10, available as separate files.

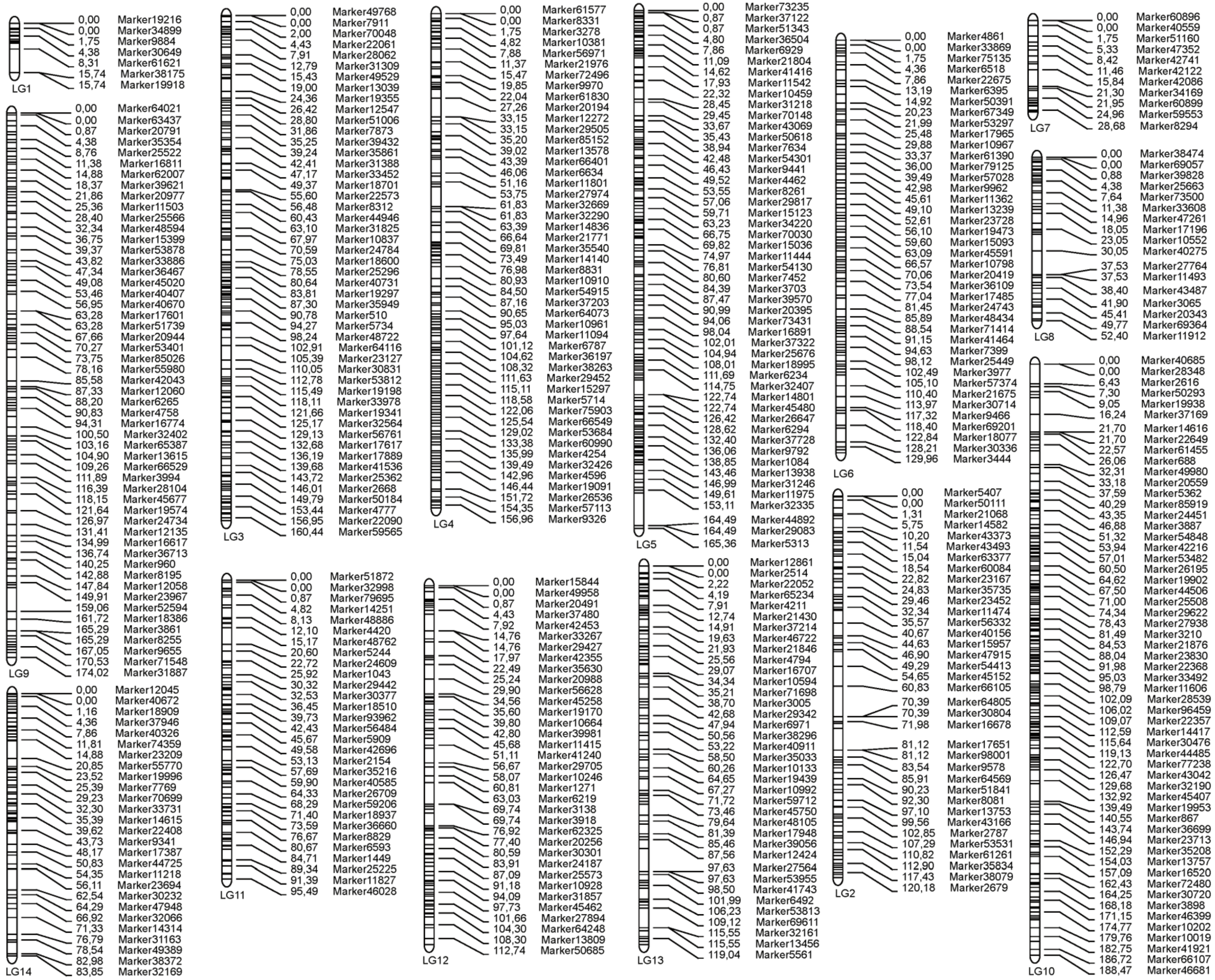

Figure S1 Sex-averaged genetic linkage group 1-14

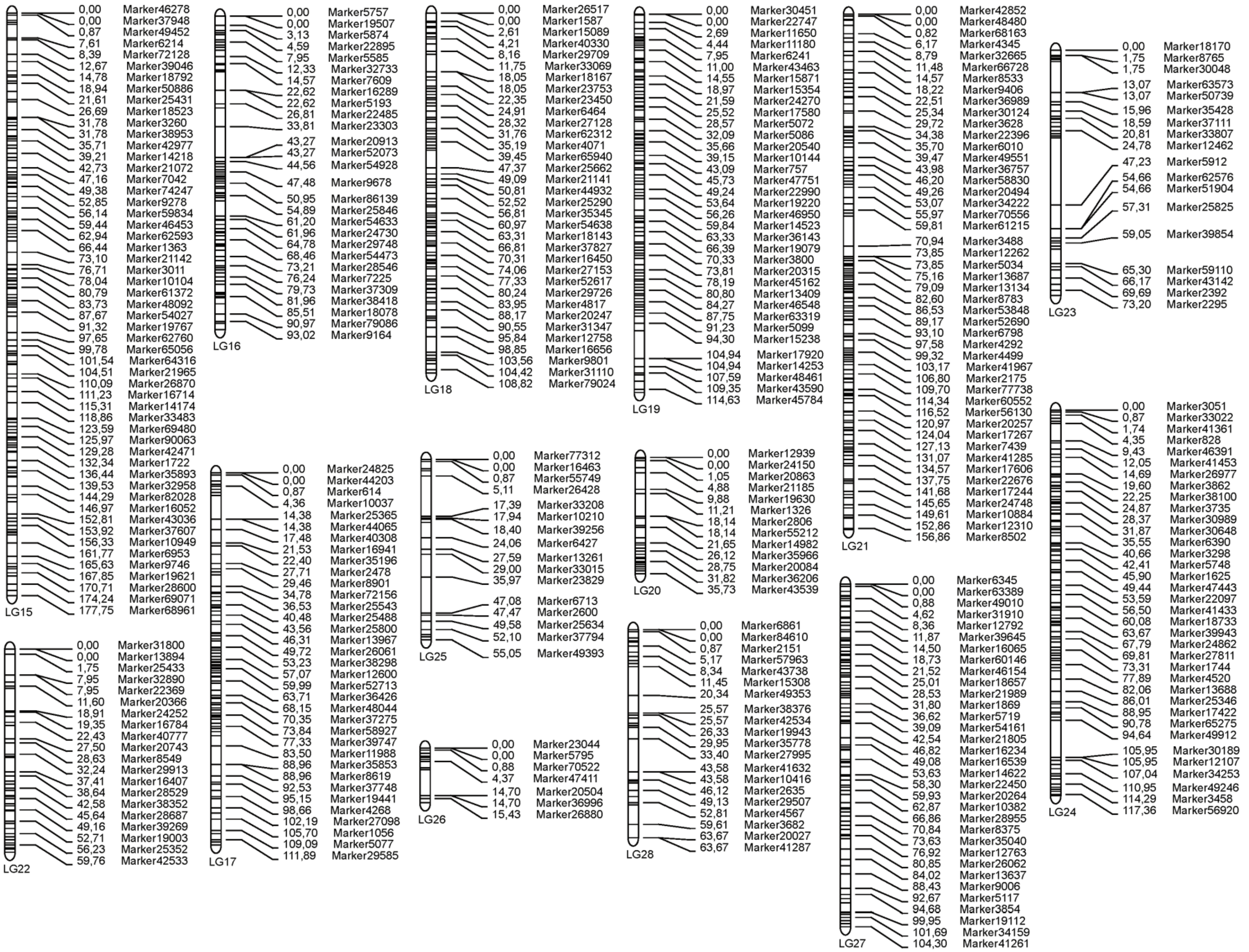

Figure S2 Sex-averaged genetic linkage group 15-28

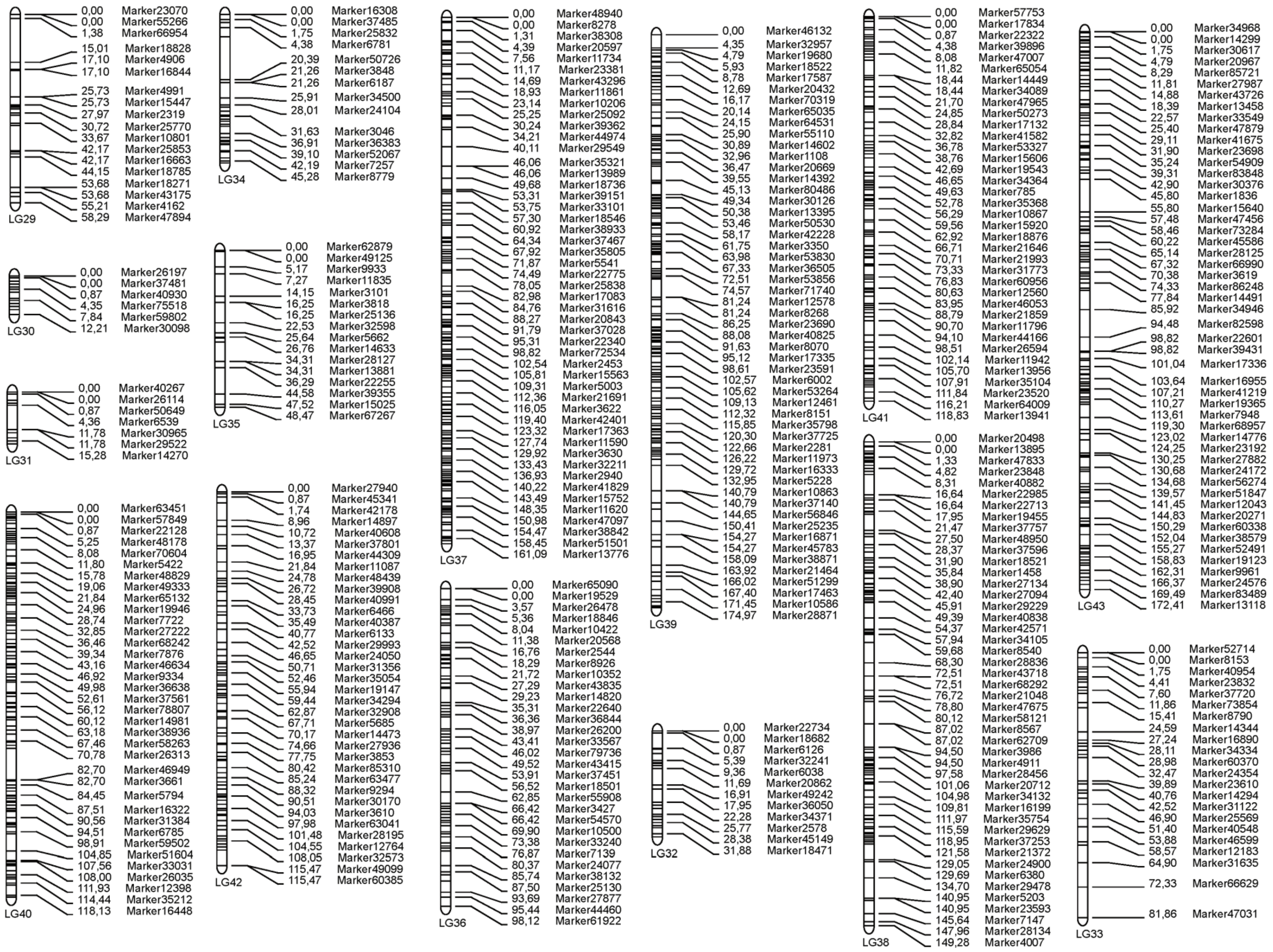

Figure S3 Sex-averaged genetic linkage group 29-43

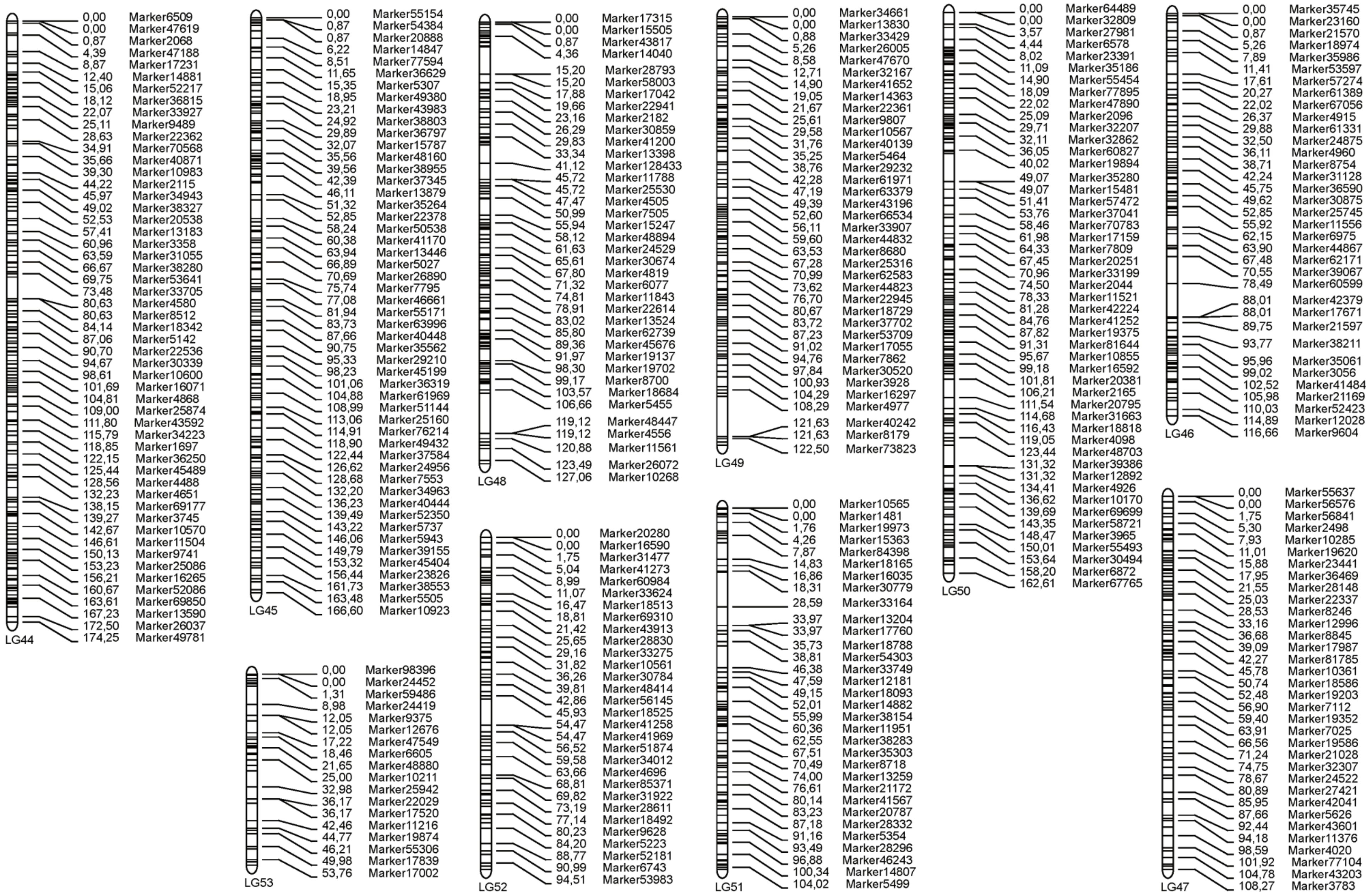

Figure S4 Sex-averaged genetic linkage group 44-53
